# Match_Motif: A rapid computational tool to assist in protein–protein interaction design

**DOI:** 10.1002/pro.4208

**Published:** 2021-10-26

**Authors:** Martin Zacharias

**Affiliations:** ^1^ Center of Functional Protein Assemblies Technical University of Munich Garching Germany

**Keywords:** binding interaction motifs, interaction design, protein design, protein–protein binding, protein–protein interface design

## Abstract

In order to generate protein assemblies with a desired function, the rational design of protein–protein binding interfaces is of significant interest. Approaches based on random mutagenesis or directed evolution may involve complex experimental selection procedures. Also, molecular modeling approaches to design entirely new proteins and interactions with partner molecules can involve large computational efforts and screening steps. In order to simplify at least the initial effort for designing a putative binding interface between two proteins the Match_Motif approach has been developed. It employs the large collection of known protein–protein complex structures to suggest interface modifications that may lead to improved binding for a desired input interaction geometry. The approach extracts interaction motifs based on the backbone structure of short (four residues) segments and the relative arrangement with respect to short segments on the partner protein. The interaction geometry is used to search through a database of such motifs in known stable bound complexes. All matches are rapidly identified (within a few seconds) and collected and can be used to guide changes in the interface that may lead to improved binding. In the output, an alternative interface structure is also proposed based on the frequency of occurrence of side chains at a given interface position in all matches and based on sterical considerations. Applications of the procedure to known complex structures and alternative arrangements are presented and discussed. The program, data files, and example applications can be downloaded from https://www.groups.ph.tum.de/t38/downloads/.

## 1 INTRODUCTION

Over the years, the database of protein structures has grown from a few atomic resolution structures in the 1980s to hundreds of thousands of entries.^1^, ^2^, ^3^ It also includes the structures of many protein–protein complexes and multicomponent assemblies.^2^ In recent years, the design of new protein structures and new protein–protein interactions to generate new proteins or complexes with a desired function has become a new research focus in protein science.^4^, ^5^, ^6^, ^7^ One powerful experimental tool for the stepwise adaptation of a protein sequence toward a desired function is the directed evolution method. It is based on random mutagenesis of a protein sequence or segment of a protein and subsequent selection according to a desired target function.^8^, ^9^ Typical applications are enzymes with known structure and function that are evolved toward new specificity and increased thermostability.^8^, ^10^ Application to design new pairwise or oligomeric interactions are also possible.^11^ However, the process requires usually repeated mutation and selection cycles and depending on the required changes the process can be time consuming and requires significant experimental efforts.

Enzyme and protein design can be supported by computational (rational) design methods. If the structure of the protein or protein–protein complex is known the effect of introducing a mutation in a protein can be studied using a variety of computational approaches.^12^, ^13^, ^14^, ^15^ Available methods range from molecular dynamics‐based free energy simulation^16^ to very simple computational scoring methods of replacing a residue based on just one structure or an ensemble of structures representing the wild type or mutated system.^14^, ^17^, ^18^, ^19^, ^20^ A few approaches are available that allow for a complete redesign of a protein based on a backbone scaffold.^4^ The most frequently used approach is based on the Rosetta program suite^21^, ^22^ and allows one to completely redesign the residues of a given backbone structure to achieve a stable folded protein using a Monte Carlo approach in combination with an appropriate scoring of the designed structures.^21^, ^23^ This method has been used in several applications to successfully redesign proteins or to create completely new stable folded proteins.^4^, ^5^, ^7^, ^23^ Combinations of rational design and directed evolution have also been used successfully to generate new or altered proteins.^9^, ^24^ In addition to the design of single proteins rational approaches have also been used to design stable protein–protein interactions.^6^, ^7^, ^25^, ^26^ A common method implemented in the Rosetta approach is based on identification of a putative interacting region, for example, a small putative interaction motif (Motifgraft), and the subsequent computational de novo design of a new protein partner around the motif and possibly additional interactions to the partner protein.^25^, ^27^ Such approach is, however, computational quite demanding and requires the computational evaluation of many in silico residue substitutions and further energy minimization and molecular modeling steps. The computational evaluation may also be of limited accuracy because of possible force field artifacts and the neglect of accounting for solvent effects in most modeling steps.

In order to simplify the initial search for a putative desired protein–protein interface the computational Match_Motif tool has been developed. The approach is entirely based on interfaces of known protein–protein complexes. The user provides a desired initial geometric arrangement of two proteins that should include a putative contact surface. It may originate from a manual placement or can be obtained as putative complex by some protein–protein docking software. The tool then searches through a database of structural interaction motifs (based on the arrangement of short backbone segments in known complexes). All motifs that structurally match to a corresponding motif at the desired interface are collected. The desired contact surface can then be re‐modeled based on the side chains of the matching motifs. The procedure takes a few seconds on a standard workstation computer. Since the final contact surface is based on motifs extracted from known favorable protein–protein interactions it likely also provides stable binding of the desired interface or at least provides a basis for further adjustment or refinement using more demanding methods. In a first part, the workflow of the method will be presented followed by several test applications to recover known highly stable interfaces or to design putative new arrangements. Additional possible applications of the tool and directions of further improvement are also discussed.

## 2 RESULTS

**FIGURE 1:**
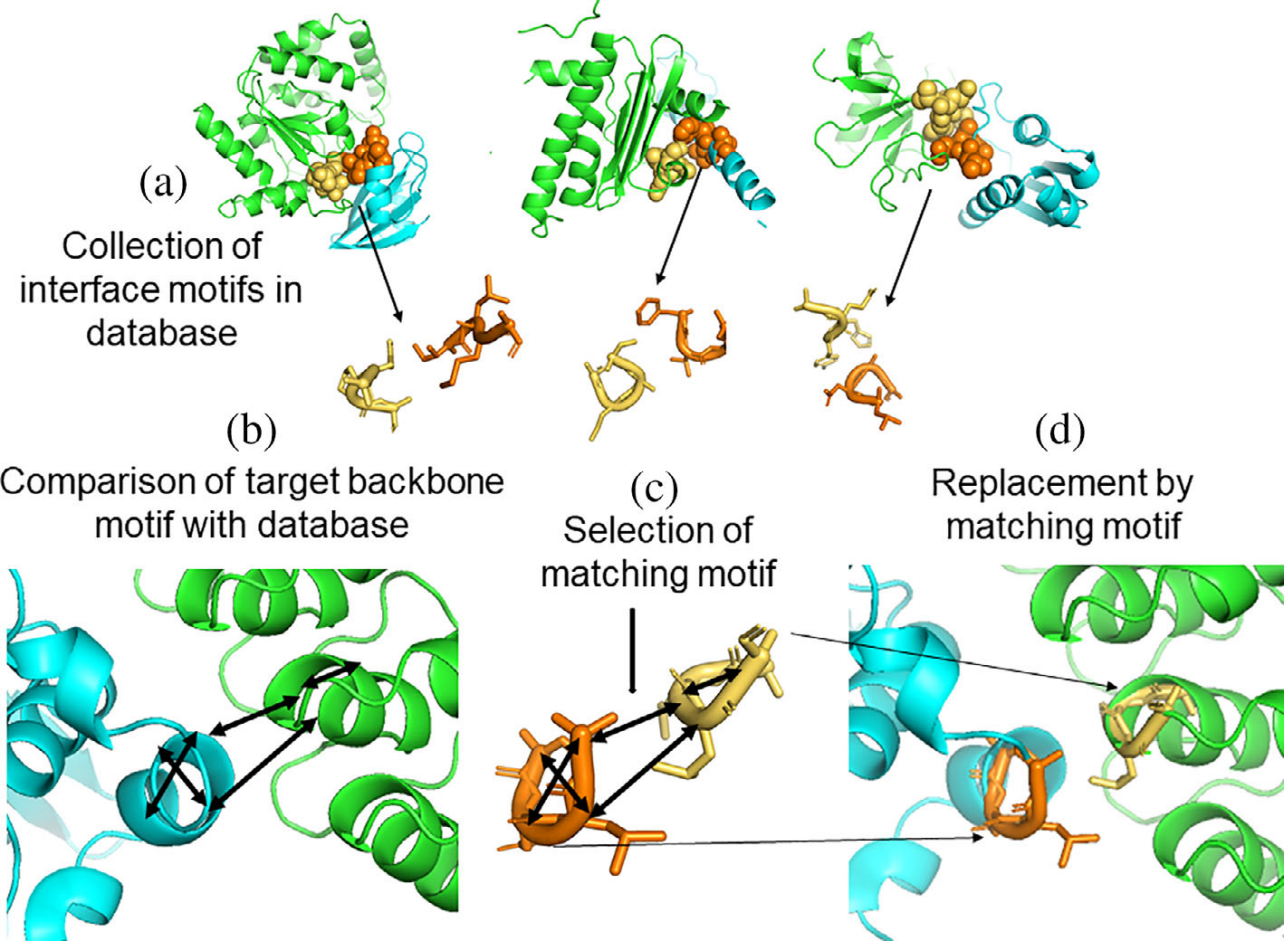
Illustration of the Match_Motif approach. (a) Structural motifs of four consecutive amino acids in each partner with a center‐of‐mass distance <10 Å are extracted from known protein–protein complexes. The interaction motifs are stored in a database and are characterized by a set of backbone (Cα‐Cα) distances. (b) The user provides a desired protein–protein interaction geometry that serves to extract putative interaction motifs (characterized by sets of distances between four residues on each partner). (c) A matching interface motif is selected. (d) The corresponding motif at the interface is replaced by the matching motif from the database providing side chains that fit sterically and allow favorable interaction in the desired geometry

For the design of assemblies of proteins with a desired function often a specific binding geometry of interacting protein partners is required. Hence, for a given pair of stable proteins that act as building blocks a stable binding in a preselected arrangement is desired. In the Match_Motif approach the user needs to provide such desired geometry as an input. Based on the backbone geometry of both partners at the interface, the algorithm searches for structural motifs of the target arrangement and searches for similar motifs in a database extracted from known stable complexes. A structural motif is here defined as four consecutive interface residues in one and another four consecutive residues in the other partner within a threshold distance of 10 Å. The motif is characterized by the distances between backbone atoms within each partner (within each of the four residues) and between the two short stretches of residues (Figure 1). The database stores ~250,000 such structural interaction motifs extracted from 1,686 structures of protein–protein complexes extracted from the PDBbind.^28^ In the following, a reduced set with ~57,000 structural interaction motifs was employed that allows one to search through all motifs within <5 s for all tested cases on a standard workstation PC (see Section 4 for details).

A match between an interaction motif in the target interface and a motif in the database is characterized by a small root mean square deviation of the backbone atom distances (dRMSD) and root‐mean‐square deviation (RMSD) of the superposition of target motif and database motif (it is typically set to <1 Å, but can be set as input parameter by the user). Once a match has been found it is stored and for constructing a new interface structure the interface segment is replaced by the database motif (only side chains are replaced that do not lead to sterical overlap with other residues at the interface). Upon completion of the algorithm, typically several side chains at the target interface are replaced by the side chains found in the matching motifs from the database. Since the side chains in the database motifs are all extracted from known stable protein–protein complexes, it is likely that the redesigned interface also forms a stable complex or at least is a good starting point for further adjustments or refinements. The process steps and workflow of the Match_Motif approach are outlined in Figure 1.

### 2.1 Application to known protein–protein complex structures

**FIGURE 2:**
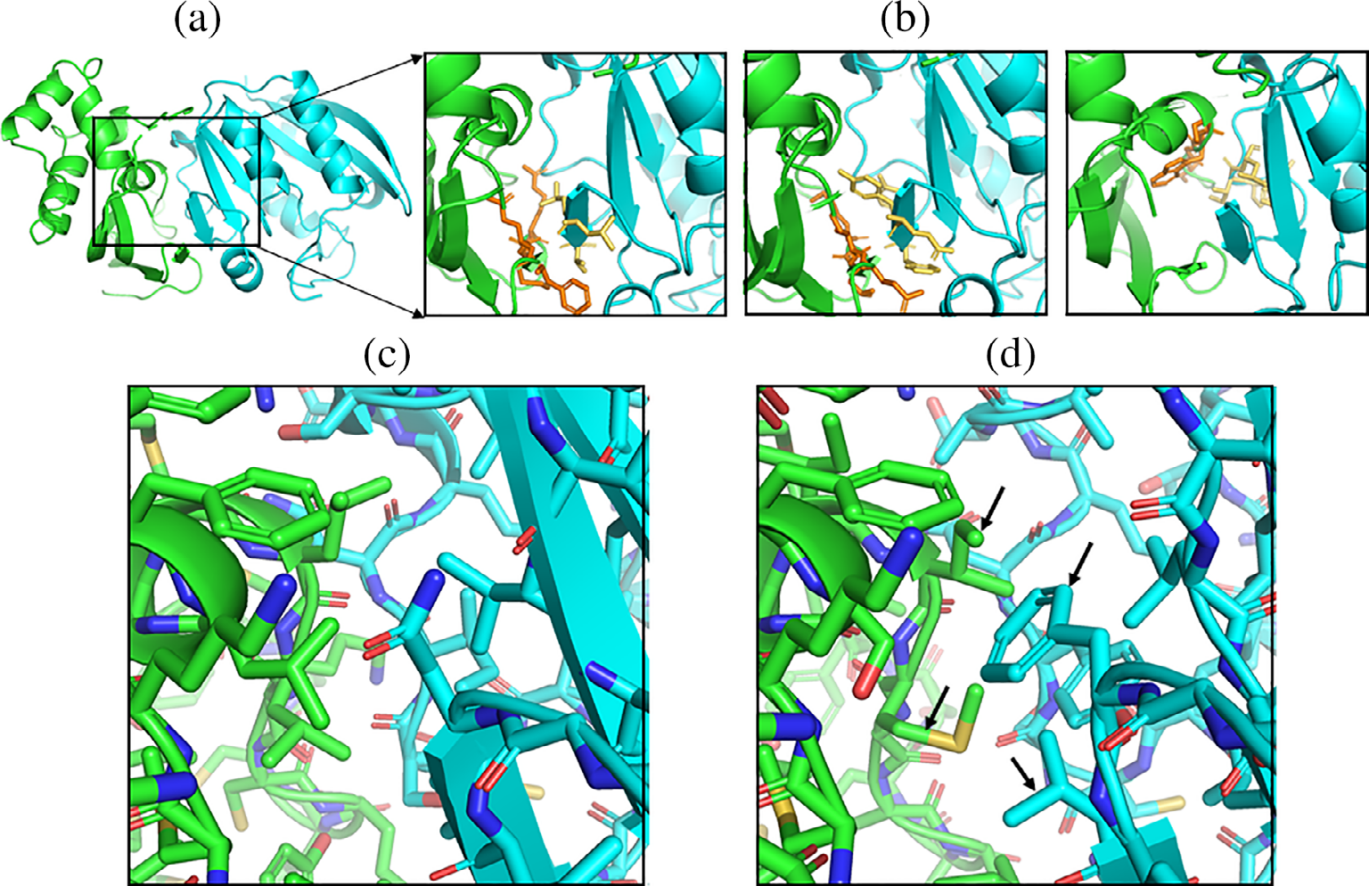
Application to the protein–protein interface of PDB 3Q87 (methyl transferase activator in complex with transferase). (a) Native complex structure as cartoon (green: chain a, blue: chain b) with the interface region indicated by a rectangle. (b) Enlarged interface region with three examples of matching alternative structural interface motifs (indicated as stick models in orange and yellow) superimposed to the matching interface segments. Each motif consists of four consecutive residues on each partner and contacting side chains extracted from a database of complex structures. Note that the view in the last panel is slightly rotated relative to the two other panels. (c) View to the native interface structure. (d) Interface structure obtained after application of the Match_Motif approach. Some side chains are replaced by side chains extracted from matching motifs (indicated by black arrows)

As a first application, the method was tested on a diverse set of 10 known protein–protein complexes with different interfaces (Table 1). The complexes were not included in the design of the database of interaction motifs. The partner proteins were placed in the native interaction geometry and for the matching procedure only target motifs with a center‐of‐mass distance between the four consecutive residues on each partner <10 Å were included. The RMSD threshold for a match was set to 1 Å. With these criteria for defining a structural match, the Match_Motif approach identified in each case several matches (Table 1). The number of matches can vary depending on the size and type of protein–protein interface (for the considered cases between ~20 and 160, Table 1). The matches may originate from homologous complexes but can also originate from complex structures that are not overall similar to the target complex structure. Many local recurrent structural interaction motifs occur in unrelated complex structures (e.g., segments of interacting α‐helices, loop–loop, loop–helix interactions, etc.). With the help of a molecule viewer such as Pymol, it is possible to easily visually inspect the structural interface matches (illustrated in Figure 2a,b for the PDB 3Q87 example case). In addition to the superimposed matching motifs, the Match_Motif approach also provides a new alternative complex structure that can include several substitutions of residues at the interface (indicates as accepted changes in Table 1). Note that the substitutions are entirely based on the collection of structural interface motifs in known complex structures. Only changes are accepted that do not result in significant sterical overlap with other residues of the partners and are selected based on the frequency of occurrence of a side chain in all matches for a given position at the interface (see also Section 4). Since the algorithm searches for interaction motifs from known complexes (that are usually well packed at the interface) and replaces a similar (backbone) matching motif at the desired interface the resulting alternative interface is typically also well packed. For example, in case of the PDB 2O25 complex with a relatively small interface, three substitutions in the buried interface region (accessible surface area, SASA, of side chains <10 Å^2^) included a Glu‐> Leu, an Arg‐> Ala, and a Thr‐> Ile substitution. In case of PDB 3Q87, with a larger interface, six substitutions at the buried interface were accepted (Leu‐> Ser, Leu‐> Met, Val‐> Leu, Thr‐> Ile, Val‐> Ala, and Ile‐> Tyr). Hence, both substitutions toward larger and sometimes smaller residues are observed such that the average size of interface residues does not change significantly. The approach in its present form does not include further (possibly costly) energetic evaluation of the substitutions. However, for the user it is possible to not only evaluate the suggested alternative interface but to inspect independently all suggested matching motifs (e.g., using a molecule viewer) and further evaluate the suggested changes using other computational tools or direct experimental testing.

**TABLE 1:** Structural motifs found by Match_Motif at interfaces of native complex structures

PDB‐ID of complex	Number of motifs^a^	Accepted changes
2o25	51	6
1syx	52	7
1t6g	31	9
1v74	57	6
1ohz	117	11
4qko	70	7
4uhp	26	10
3e8l	90	7
3q87	161	19
2w57	19	5
1ppe	110	25(11)
1udi	98	14(9)

^a^ Matching motifs were identified in a collection of 57,000 motifs (not including the 10 test complexes, first 10 rows) with a backbone RMSD threshold of 1 Å. The last two rows indicate application to two complexes that were included in the motif database generation. For these cases numbers in parenthesis indicate the accepted motifs using a backbone RMSD threshold of 0.25 Å (all accepted motifs reproduce the original interface sequence).

As a further test, the approach was also applied to two complexes that were also used to build the database of interface motifs (last two rows in Table 1). With the standard settings (with 1 Å backbone RMSD threshold for a match), several alternative matching motifs were found in these cases resulting also in a few substitutions at the interface. This is expected since for these examples, the database can contain matching motifs from other complexes. However, if the RMSD threshold for a match is sufficiently lowered (to 0.25 Å) exactly the residues found in the interface of the known complex are recovered (the match from the database corresponds to a motif from the same complex structure or a very close homolog!).

### 2.2 Effect of small changes in the partner arrangement

**FIGURE 3:**
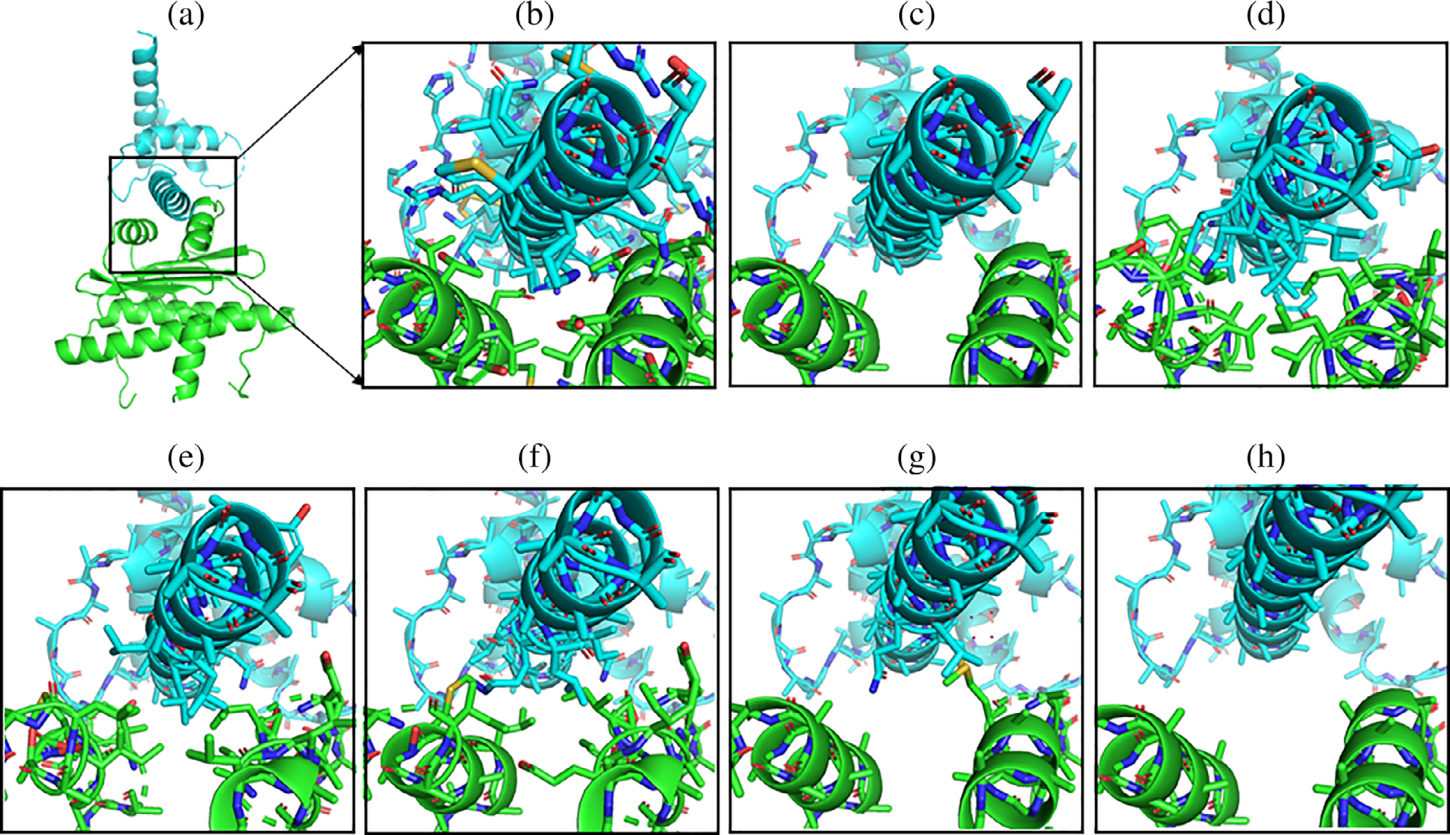
Search for interface motifs upon distance change between partner proteins. (a) Protein–protein complex PDB 3P57 with the interface region indicated by a black rectangle. (b) Native interface structure in stick representation. (c) Native interface with all side chains removed as start for the Match_Motif application. Rebuilt interface structure after application of Match_Motif. Note that the interface side chain structure differs at several positions from the native interface structure (shown in b) because Match_Motif identifies alternative putative interface residues occurring more frequently in the matching motifs. (e, f) Generated interface structure for different distances of the partner proteins relative to the native placement. The distance of the blue from the green partner was increased in steps of 1 Å (e), 2 Å (f), 3 Å (g) to up to 4 Å (h). For the case (e), an interface structure similar to the generation for the native placement (in d) was obtained but no matching motif was returned for the displacement by 4 Å (h)

As a next step, the sensitivity of the approach to small changes of the placement of the partners relative to the native binding geometry was tested. For three test cases (Table 2), one partner protein was moved along a line connecting the center‐of‐mass of the interface atoms of each partner (atoms within 10 Å of the other partner) in steps of 1 Å. All residues of the protein partners were replaced by Ala residues and matching interface motifs were collected with the Match_Motif approach (Table 1). Due to the stepwise separation of the partners, a larger cutoff for atoms belonging to the interface (12 Å) in addition to the default of 10 Å was also used. This leads to overall more matches but as expected the number of matches decreases rapidly with increasing distance between protein partners (Table 1 and illustrated for the PDB 3P57 case in Figure 3). Already at displacements along the separation coordinate by 3 Å very few or no matches are found. The result is not unexpected since the distance between protein partners is critical for obtaining a sterical possible but also well‐packed interface. If the distance becomes too large, there are simply no interaction motifs available that still include side chains in close contact with each other. It also indicates that the user needs to place the two protein partners fairly precisely (to within 1–2 Å) in order to obtain a reasonable number of interface structural motifs. However, since the application of the Match_Motif approach requires only a few seconds it is in principle possible to rapidly test slightly different interface geometries and compare the packing and stereochemistry of the obtained interface matches (see below).

**TABLE 2:** Partner–partner distance dependence of Match_Motif application

Complex	3P57		1AY7		1HE1	
Distance (Å)^a^	12.0/1.0^b^	10.0/1.0	12.0/1.0	10.0/1.0	12.0/1.0	10.0/1.0
0.0	614	172	52	20	200	104
1.0	386	66	13	8	184	53
2.0	182	0	8	7	67	17
3.0	28	0	11	4	22	3
4.0	0	0	2	0	3	0

Abbreviation: RMSD, root‐mean‐square deviation.

^a^ Distance indicates the distance from the native geometry along a line connecting the center‐of‐mass of the interface regions on both partner proteins.

^b^ For each case, two searches were performed, one with the standard setting for the maximum distance between partner segments of 10 Å and counting all matches with backbone RMSD <1.0 Å (10/1.0); in the other, the maximum segment distance of 12 Å was used (12.0/1.0).

### 2.3 Design of putative protein–protein interfaces for example cases

**FIGURE 4:**
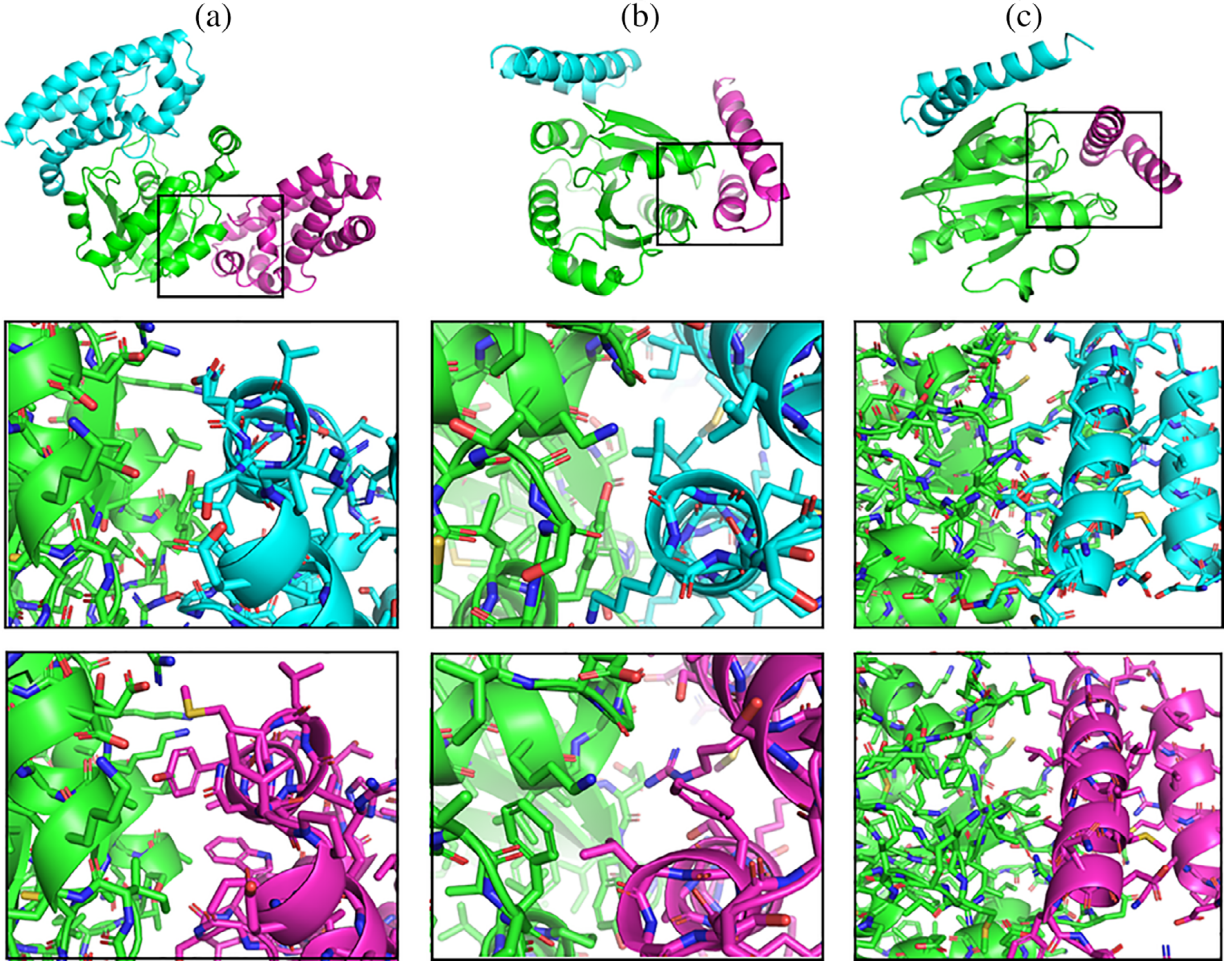
Examples of redesigned interfaces. (a) The top panel shows the native protein–protein complex PDB 1J2J (blue and green cartoon) and an alternative arrangement (magenta) obtained from a docking run using the ATTRACT program ranked as top100 solution (native complex ranked as top1). The interface region is indicated as black rectangle. In the second row, the enlarged interface at the alternative docking solution (top100, green and blue partner structures), and in the third row the redesigned interface using the Match_Motif approach is shown (here, the second partner is shown in magenta). (b) Same as (a) but for the complex PDB 1HE1 (an alternative docking solution scored as top94 was used). (c) Same as (b) but using a manually placed alternative arrangement for the second partner (magenta)

One possibility to generate alternative protein–protein interaction geometries at a reasonable distance of the partner proteins to yield a sufficient number of matching motifs is to apply a protein–protein docking approach. Such docking approach typically results in near‐native solutions but also in a large number of alternative binding geometries that may not score as well as the near native solutions. In the present case, the ATTRACT docking program^29^, ^30^, ^31^ was used to obtain alternative binding geometries for the PDB 1HE1 complex (using rigid partner proteins). In a systematic docking run the surface of the protein partners is systematically scanned for potential interaction geometries usually resulting in thousands of local energy minimum solutions. For the PDB 1HE1 case, the solution ranked at position 100 (top100 solution) significantly differs from the native geometry (Figure 4a) with an interface RMSD (IRMSD) = 20.8 Å and a ligand RMSD (LRMSD) = 54 Å. The IRMSD measures the RMSD of all atoms that belong to the native interface in the top100 complex relative to the same atoms in the native complex. The LRMSD measures the RMSD of one partner protein relative to the native placement after best superposition of the second protein (receptor) onto the receptor in the native complex. The ATTRACT program calculates for each docked complex a knowledge‐based score in RT units (R: gas constant, T: temperature, RT unit indicates the mean energy per degree of freedom at a temperature T). For the native complex a score of −20.1 RT units (top1 solution) was obtained and −12.2 RT units for the top100 solution. The top100 solution does not score well because of several unfavorable polar–nonpolar contacts at the alternative interface and nonoptimal packing (Figure 4a, second row). Nevertheless, if we consider this geometry as a desired geometry one can use the Match_Motif approach to identify putative matching interface motifs for the top100 geometry. Indeed, the alternative complex structure obtained as output of the program contains several residue substitutions. The structure was energy minimized to remove any residual sterical overlap (see Section 4) and the redesigned partners were used as input for another systematic ATTRACT docking run. In this case, docking solutions very close to the new (top100‐based) geometry were obtained that scored with an ATTRACT score of −18.8 RT units as “new” top1 solution. It also stayed close to the (old) top100 solution with a deviation from the (old) top100 geometry of IRMSD = 1.4 Å and LRMSD = 2.4 Å (Figure 4a, lower panels). Hence, at least the in silico analysis of the redesigned interface predicts a significant improvement by the Match_Motif approach.

The same procedure was also tested on a docking solution for the complex PDB 1J2J (taking a solution that ranked as top94 solution in a systematic docking run with an ATTRACT score of −9.1 RT, compared to −15.1 RT for the native solution). Here, the interface redesign resulted in an alternative complex structure that upon systematic redocking gave a score of −13.4 RT units and scored at position top9 (Figure 4b) again a significant predicted improvement of the new interface.

Finally, a manual placement of the partners at another putative interaction geometry (illustrated in Figure 4c) was also tested. The manual docking placement was achieved using the Pymol software^32^ and checking of the protein–protein distance in the edited placement. The desired interface contains several charged and polar residues and not necessarily favorable contacts to nonpolar residues (Figure 4c) resulting in an ATTRACT score of −5.6 RT units. The Match_Motif approach generated an alternative complex interface structure that upon systematic docking scored as top32 solution with an ATTRACT score = −13.1 RT units.

**FIGURE 5:**
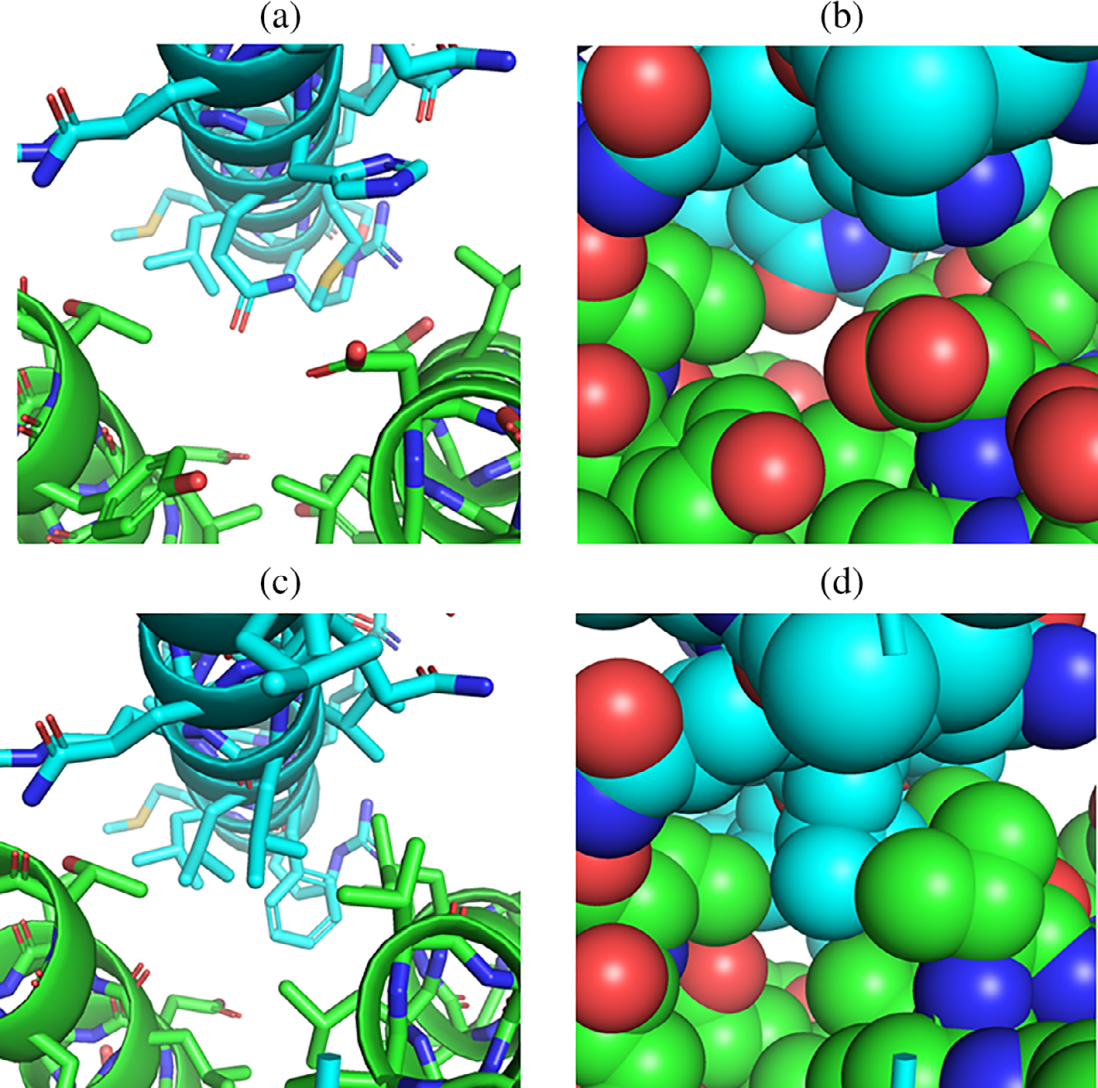
Optimization of partner protein placement. (a) An initial manual and nonoptimal placement of an α‐helical partner (blue) relative to a putative partner (green) gives only few interface matches. (b) The nonoptimal packing at the interface is illustrated by a van der Waals representation of the interface. (c) After optimization of the placement in (only) translational steps of 1 Å a more hydrophobic and sterically better packed interface is obtained by the Match_Motif approach (better van der Walls packing illustrated in (d))

As indicated already in the previous paragraph, a manual placement of protein partners may not yield an optimal number of matching interface motifs (e.g., because in case of a too large or too small distance between partners no motifs that yield reasonable side chain contacts and/or no residue overlap are found in the motif database). Such case is illustrated in Figure 5a for an α‐helix (blue in Figure 5a) that is desired to bind at the space between two helices of the second partner (green in Figure 5a). At the starting placement, no substitutions of interface residues are suggested and the interface is not well packed (Figure 5b). A systematic shifting of the single helix relative to the partner protein in all spatial directions yields one placement that moves the ligand protein closer to the partner protein (by ~1 Å) resulting in several matches that produce an alternative interface structure with several hydrophobic residues better packed than the initial geometry (Figure 5c,d).

## 3 DISCUSSION

The large collection of proteins and protein–protein complexes with known structure forms a rich basis of extracting recurrent motifs at protein–protein interfaces. Such local motifs of contacting consecutive residues on both partner proteins occur not only in homologous complexes but can also form locally as part of interfaces in unrelated complexes. The Match_Motif approach rapidly compares all backbone interaction motifs at a desired interface with the known available motifs with sterically well‐fitting intermolecular contacts. For a target interface of sufficient size that places both proteins at a reasonable distance to form a stable complex typically in the order of ~100 structural interface motifs can be found. The process requires only a few seconds but could be further accelerated by a smarter algorithm to search through available motifs. In the future, this could be achieved for example by first searching through a structural classification of motifs that contains only a few representatives followed by an extended search through only the members of a few matching classes.

It should be emphasized that the present approach can be used to redesign a known interface by suggesting appropriate substitutions. However, in such a case, it may also be useful to directly search for evolutionary conserved related complexes^33^ in order to identify suitable residue substitutions. Even in such case, the Match_Motif approach can suggest appropriate substitutions if a structure of such related complex is available and used to provide motifs stored in the database. However, a desired focus of the Match_Motif approach is to offer the possibility to find appropriate matches even for a new interface with no evolutionary conserved homologs. Besides of a collection of matching motifs, the approach also provides a newly generated interface structure with new interface side chains extracted from the matching motifs. The selection of the side chains is solely based on frequency of occurrence in the matching motifs and sterical considerations. For the example cases, the algorithm resulted in changes that considerably improved the scoring of the redesigned interface based on a knowledge‐based scoring function. This is a good hint but no guarantee that the exchanges at the interface indeed improved the binding affinity. In this regard, it is important to emphasize that the purpose of the Match_Motif program is not to automatically provide a new stable interface for every desired binding arrangement of two proteins. It can, however, be useful to provide a starting point for further refinement and to exclude certain arrangements that do not give any match to a structural motif found in know stable protein–protein complexes. Further refinement indicates here that not all residue exchanges at a desired interface need to be tested but only a few positions of an interface that contains already residues extracted from close motifs found in other stable protein–protein complexes. The number of structural interface motifs could also be significantly extended by considering sequence homologs of known protein–protein complexes that likely form the same protein–protein complex structure as the known complex. In addition, the motif database could be further improved by distinguishing between interaction motifs that correspond to binding hotspots in known complexes and may contribute also high affinity upon copying such motifs to matching segments in desired interfaces.

## 4 MATERIALS AND METHODS

### 4.1 Extracting protein–protein interaction motifs

In the present study, an interaction motif is considered as four consecutive residues in one protein partner and four consecutive residues below a distance threshold in the second partner. The distance threshold needs to be large enough to include all interaction motifs such that residues in one four‐residue segment are in contact with residues in the four‐residue segment in the partner protein. A test with different threshold distances indicated that for distances larger than 11 Å, an increasing number of motifs are found that have no or only little contact between residues of the partner proteins. On the other side for threshold distance smaller than 11 Å, some motifs with large interacting side chains are excluded from the database. Hence, with a 11 Å cutoff structural motifs were extracted from a set of 1,686 protein–protein complexes extracted from the PDBbind database.^28^ Structures with an interface of <1,000 Å^2^ or one partner containing <40 residues were not included (the list of PDB‐IDs is included in the download package, see below), it resulted in 255,000 interface motifs. The coordinates of each motif and the distance between all pairs of Cα backbone atoms are stored in the database. A second database with a slightly reduced set of structural motifs was generated by introducing a cutoff for the mean of Cα‐Cα distance deviations (dRMSD) between motifs. A motif was only retained if it deviates from all other motifs by a sum of Cα‐Cα distances larger than the cutoff of 1 Å. Second, the set was further reduced by including only interface motifs with at least three nonpolar or aromatic residues. The reduced set contains ~57,000 motifs and allows rapid selection and was used for all applications in the present study.

### 4.2 Matching interaction motifs to interface segments

For a given protein–protein complex, the program Match_Motif identifies the interface regions of the protein partners. The interface region typically includes all residues within a distance threshold for interatom pairs between the protein partners (default is 10 Å). Similar to the collection of interface motifs extracted from known protein–protein complexes in a next step, all segments of four consecutive residues in one partner and four residue segments in the second partner within a center‐of‐mass distance below a threshold (typically 10 Å) are collected. The collected motifs are compared with all the motifs in the interaction motif database with respect to the backbone dRMSD. All structural motifs from the database below a dRMSD threshold are considered as possible matches and can replace the corresponding interface segment (with a different set of side chains but only small difference in the protein backbone). As a final check the RMSD of the target motif (backbone) relative to the database motif is calculated and used for selection (with a default threshold of 1 Å). With this threshold for a protein–protein interface (of a known complex) with typical size between 20 and 200 matches can be collected. The matching segments are stored in a separate file and can be inspected visually. However, the program also outputs a PDB‐file with an alternative interface based on the matching motifs. An interface residue is replaced by the most frequently found residues for a given position found in all matching motifs if it does not overlap with other residues in the partner proteins. To remove residual overlap and optimize the alternative interface arrangement an energy minimization employing a molecular mechanics program is performed. For the present cases, it was performed using first the *sander* program of the Amber18 package^34^ employing an implicit solvent model with a distance dependent dielectric constant (4r) and 400 steps of energy minimization followed by energy minimization using a Generalized Born implicit solvent model^35^ (igb = 5 option in Amber, 2,000 steps) and the *pmemd*.*cuda* module of Amber18. The FF14SB force field^36^ was used during all minimization steps.

The Match_Motif program, a manual, and the data file of structural interface motifs as well as example applications can be downloaded from https://www.groups.ph.tum.de/t38/downloads/.

## AUTHOR CONTRIBUTIONS

**Martin Zacharias**: Conceptualization, investigation, writing, resources, funding acquisition.
